# Supplementary material for: A Manual of Procedures for the Generation of the AI-Ready and Exploratory Atlas for Diabetes Insights (AI-READI) Database
Source: medRxiv. 2026 Apr 4:2026.03.30.26349552. Preprint. [Version 1] doi: 10.64898/2026.03.30.26349552 (PMC13060450; doi:10.64898/2026.03.30.26349552)
Supplement: 1 [file NIHPP2026.03.30.26349552V1-supplement-1.pdf]

medRxiv preprint doi: https://doi.org/10.64898/2026.03.30.26349552; this version posted April 4, 2026. The copyright holder for this preprint (which was not certified by peer review) is the author/funder, who has granted medRxiv a license to display the preprint in perpetuity.
It is made available under a CC-BY 4.0 International license .

20. Appendix

The following pages contain AI-READI-specific documents that support recruitment, protocol details, return of data and data interpretation.

medRxiv preprint doi: https://doi.org/10.64898/2026.03.30.26349552; this version posted April 4, 2026. The copyright holder for this preprint (which was not certified by peer review) is the author/funder, who has granted medRxiv a license to display the preprint in perpetuity.
It is made available under a CC-BY 4.0 International license .

APPENDIX A. Recruitment Letter

UAB MEDICINE
UC San Diego Health

AI-READI
AI Ready and Exploratory Atlas for Diabetes Insights

UW Medicine
UW SCHOOL OF MEDICINE

Dear [X],

Did you know that an estimated 33 million people are living with type 2 diabetes in the United States?
While type 2 diabetes typically develops in adults over the age of 45, there are increasing numbers of children, teenagers, and young adults diagnosed each year. AI-READI is a research program about type 2 diabetes.

Why are we contacting you?

- We are gathering health data from people **with and without** type 2 diabetes, to create a dataset that will be used by scientists to improve our understanding of type 2 diabetes and other areas of health using artificial intelligence.
- This research program is important because we are collecting many types of health data from people living in the United States to help research in improving our health.
- We obtained your contact information from your medical records. You were chosen because you live near one of our 3 research sites (University of Alabama at Birmingham, U.C. San Diego, or University of Washington).

What are the benefits to participating in AI-READI?

- We will provide you with reports of your continuous glucose (blood sugar) monitoring
- Your blood pressure, heart rate, and vision testing data will be given to you at your visit
- As a token of our appreciation, you will receive $200 (on an electronic gift card) for your full participation
- By contributing health data, you would be helping scientists better understand type 2 diabetes and overall health

What are we asking you to do?

At home:	One visit to our research site (~3-4 hours):	10 days at home:
Complete Questionnaires	Physical Assessment; Cognitive Test; ECG (heart activity); Testing your sense of touch; Vision Testing (eye charts); Retinal Imaging (pictures of the backs of your eyes); Blood and Urine Samples	Continuous Blood Sugar Monitor; Physical Activity Monitor; Environmental Sensor (air, light, temperature, etc.)
	*Free Parking (or reasonable transportation, if needed)	Ship devices back (we pay)

You receive $200 when devices are returned

Would you like to learn more about the AI-READI research program?

- Use this link to take you to the next steps: https://redcap.iths.org/surveys/ or
- **Your personal access code**: [XXXXX]

We are also happy to answer your questions by phone or email. Here's how to contact us:

- Living in the Birmingham area: (205) 297-0408, or email us at ai-readi@uabmc.edu
- Living in the San Diego County area: (858) 822-4669, or email us at ai-readi@health.ucsd.edu
- Living in the Seattle area: (206) 710-6689, or email us at ai-readi@uw.edu

medRxiv preprint doi: https://doi.org/10.64898/2026.03.30.26349552; this version posted April 4, 2026. The copyright holder for this preprint (which was not certified by peer review) is the author/funder, who has granted medRxiv a license to display the preprint in perpetuity.
It is made available under a CC-BY 4.0 International license .

APPENDIX B: Participant Return of Data Card

Thank you for taking part in the AI-READI research study. Your information will provide scientists with data that could lead to a better understanding of type 2 diabetes. We would like to share some of the measurements we collected from you today. Below, please find your blood pressure, heart rate, and vision data.

Date of Visit: ______________________________

Blood Pressure (Systolic / Diastolic): _______ / _______

Resting Heart Rate; Beats Per Minute (BPM): _________

Visual Acuity; Right Eye: Snellen 20 / _______

Visual Acuity; Left Eye: Snellen 20 / _______

☐ Your measurements today suggest a potential concern with your blood pressure or heart rate. Please see the charts below for ranges in values. We recommend that you schedule a visit with a health care provider to share this information as soon as possible.

Blood Pressure Categories

American Heart Association.

BLOOD PRESSURE CATEGORY	SYSTOLIC mm Hg (upper number)		DIASTOLIC mm Hg (lower number)
NORMAL	LESS THAN 120	and	LESS THAN 80
ELEVATED	120-129	and	LESS THAN 80
HIGH BLOOD PRESSURE (HYPERTENSION) STAGE 1	130-139	or	80-89
HIGH BLOOD PRESSURE (HYPERTENSION) STAGE 2	140 OR HIGHER	or	90 OR HIGHER
HYPERTENSIVE CRISIS (consult your doctor immediately)	HIGHER THAN 180	and/or	HIGHER THAN 120

Normal Resting Heart Rate Range (BPM)

60-100 BPM

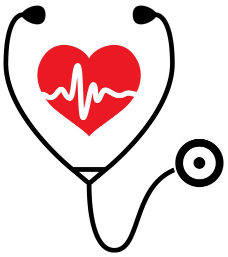

* These are guidelines for "normal" ranges of blood pressure and heart rate for adults. Your "normal" may be different for many reasons, including your age, level of fitness, and general health.

Visual Acuity:

If your visual acuity in either eye is Snellen 20/40 or worse, we suggest that you schedule an appointment for a comprehensive eye examination with an ophthalmologist or optometrist (if you have not had an eye examination during the past year). For persons with type 2 diabetes, the American Academy of Ophthalmology recommends that you have an annual screening at the time of diagnosis and at least yearly screenings thereafter.

Monofilament Testing:

During your visit today, a 10-g monofilament test for peripheral neuropathy was also performed. The American Diabetes Association recommends that all persons with type 2 diabetes undergo annual comprehensive foot evaluations, including 10-g monofilament testing.

medRxiv preprint doi: https://doi.org/10.64898/2026.03.30.26349552; this version posted April 4, 2026. The copyright holder for this preprint (which was not certified by peer review) is the author/funder, who has granted medRxiv a license to display the preprint in perpetuity.
It is made available under a CC-BY 4.0 International license .

APPENDIX C:

Please include this completed sheet in your return box!

DEVICE INFORMATION

**Please return all three devices on the date written:**	You may stop wearing all devices on: ____________________
**Please circle the number of days you were able to wear the Dexcom G6 Continuous Glucose Monitor on your skin:**	1 2 3 4 5 6 7 8 9 10
**Please write down where you placed the environmental sensor in your home:**	____________________
**Please indicate which wrist you wore the Garmin watch.**	☐ **LEFT** ☐ **RIGHT**
**Which is your dominant hand?**	☐ **LEFT** ☐ **RIGHT** ☐ **BOTH**

REMOVING DEVICES

**DEXCOM G6 CGM**	Remove the CGM by following the removal instructions provided and place the CGM in the bubble wrap sleeve.
**ENVIRONMENTAL SENSOR**	Unplug the environmental sensor and place the sensor in the bubble wrap sleeve.
**GARMIN WATCH**	Remove the Garmin watch from your wrist and place it in the bubble wrap sleeve.

RETURNING DEVICES

Place **Dexcom G6 CGM transmitter** in the box.	☐
Place the **Garmin Watch** in the box.	☐
Place the **charger for the Garmin Watch** in the box.	☐
Place the **Environmental Sensor** in the box.	☐
Place **power cable for the Environmental Sensor** in the box.	☐
Peel off the **FedEx label** provided and place it on the box.	☐
Seal the box and drop it in any **FedEx dropbox** (not a store!)<br>You may find a dropbox near you at:<br>www.fedex.com/en-us/shipping/dropbox.html	☐

Or schedule a pick-up at home by calling **1-800-463-3339** or **1-800-GoFedEx**.

If you have question or concerns during the 10 days for any devices, please email ai-readi@uabmc.edu or call 205-297-0408

medRxiv preprint doi: https://doi.org/10.64898/2026.03.30.26349552; this version posted April 4, 2026. The copyright holder for this preprint (which was not certified by peer review) is the author/funder, who has granted medRxiv a license to display the preprint in perpetuity.
It is made available under a CC-BY 4.0 International license .

APPENDIX D:

Understanding Your Continuous Glucose Monitoring Report

Thank you again for your participation in AI-READI. Along with this guide, you should have received a detailed report of your continuous blood glucose levels for the 10-day monitoring period. This document provides some guidelines for interpreting your blood glucose results.

Our research program uses the Dexcom G6 Pro Continuous Glucose Monitor (CGM). The reports of your data were generated by Dexcom Clarity. If you would like more information about how the Dexcom CGM works, you may watch a video tutorial here: https://www.youtube.com/watch?v=PRSKJoUbQmc

We provide basic information on reading your report below. More detailed information on each type of report is covered in this video tutorial (some information in the video may not apply to you): https://www.youtube.com/watch?v=znL4ftiZFOs

If you need additional help interpreting your Dexcom report and/or have questions about your results, please contact your primary health provider.

Dexcom CGM Clarity Results Overview

(go to page 3 of your CGM report for your results)

Result Example	Explanation of results
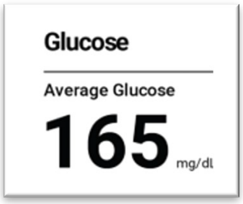	**Glucose Level**<br>This number is your average continuous blood glucose level. The number is an average of all measurements taken during your monitoring period, including all high, normal, and low glucose levels.
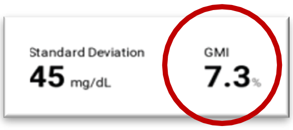	**Glucose Management Indicator (GMI)**<br>Your GMI is the average (mean) glucose value based on the data collected by the CGM. This number will be used to estimate your average A1C%.
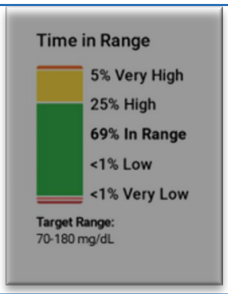	**Time in Range**<br>The time in range tells you the percent (%) of time you spent in and out of the target range for blood glucose levels during your monitoring period.

medRxiv preprint doi: https://doi.org/10.64898/2026.03.30.26349552; this version posted April 4, 2026. The copyright holder for this preprint (which was not certified by peer review) is the author/funder, who has granted medRxiv a license to display the preprint in perpetuity.
It is made available under a CC-BY 4.0 International license .

What Should You Be Looking For In Your CGM Report?

Low Blood Glucose:

1. If you take insulin, or a pill that makes your body release more insulin, you may be at risk for low blood glucose. When you lose weight or make healthier food choices, you may need less of your diabetes medication.

2. Symptoms of low glucose include:
   - Shakiness
   - Sweating
   - Confusion
   - Feeling weak
   - Feeling hungry

   If you feel these or any other unusual symptoms, you should complete a fingerstick test with your glucose meter.

3. To treat low glucose, you should eat/drink something with about 15 grams of carbohydrates/sugar. This might include ½ cup of juice, one cup of skim milk, or 3 to 4 glucose tablets. It will take at least 15 minutes for these sugars to work. If your glucose is still less than 70 mg/dL after 15 minutes, treat with another 15 grams of sugar.

Blood Glucose ≤ 70 mg/dL	Have 15 grams of straight carbs	Wait 15 minutes	Re-check blood glucose	Repeat process if still ≤ 70 mg/dL

4. If you have readings less than 70 mg/dL, you should talk with your health care provider. Ask if you should reduce your medication dose to prevent low glucose.

Diabetes medications with the greatest risk for low blood glucose:
Insulin, Glipizide, Glimepiride, Glyburide

medRxiv preprint doi: https://doi.org/10.64898/2026.03.30.26349552; this version posted April 4, 2026. The copyright holder for this preprint (which was not certified by peer review) is the author/funder, who has granted medRxiv a license to display the preprint in perpetuity.
It is made available under a CC-BY 4.0 International license .

Pay Attention to Low and High Blood Glucose Levels

Glucose Thermometer

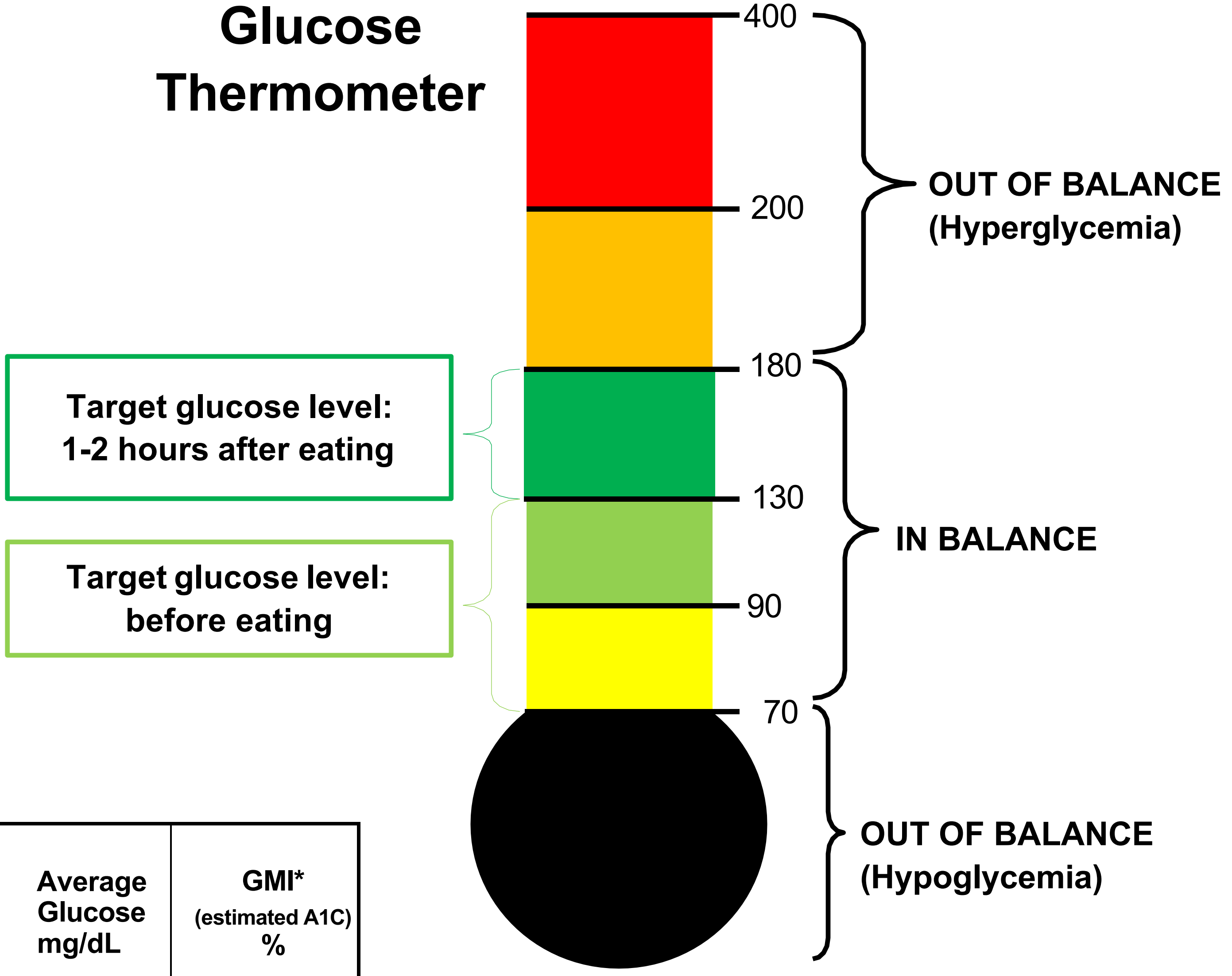

Average Glucose mg/dL	GMI* (estimated A1C) %
100	5.7
150	6.9
200	8.1
250	9.3
300	10.5

For values in the RED range, please see a health care provider as soon as possible!

**Glucose Management Indicator (GMI)*
You may find your GMI value on page 3 of your CGM Report

medRxiv preprint doi: https://doi.org/10.64898/2026.03.30.26349552; this version posted April 4, 2026. The copyright holder for this preprint (which was not certified by peer review) is the author/funder, who has granted medRxiv a license to display the preprint in perpetuity.
It is made available under a CC-BY 4.0 International license .

Example Continuous Glucose Monitoring Report

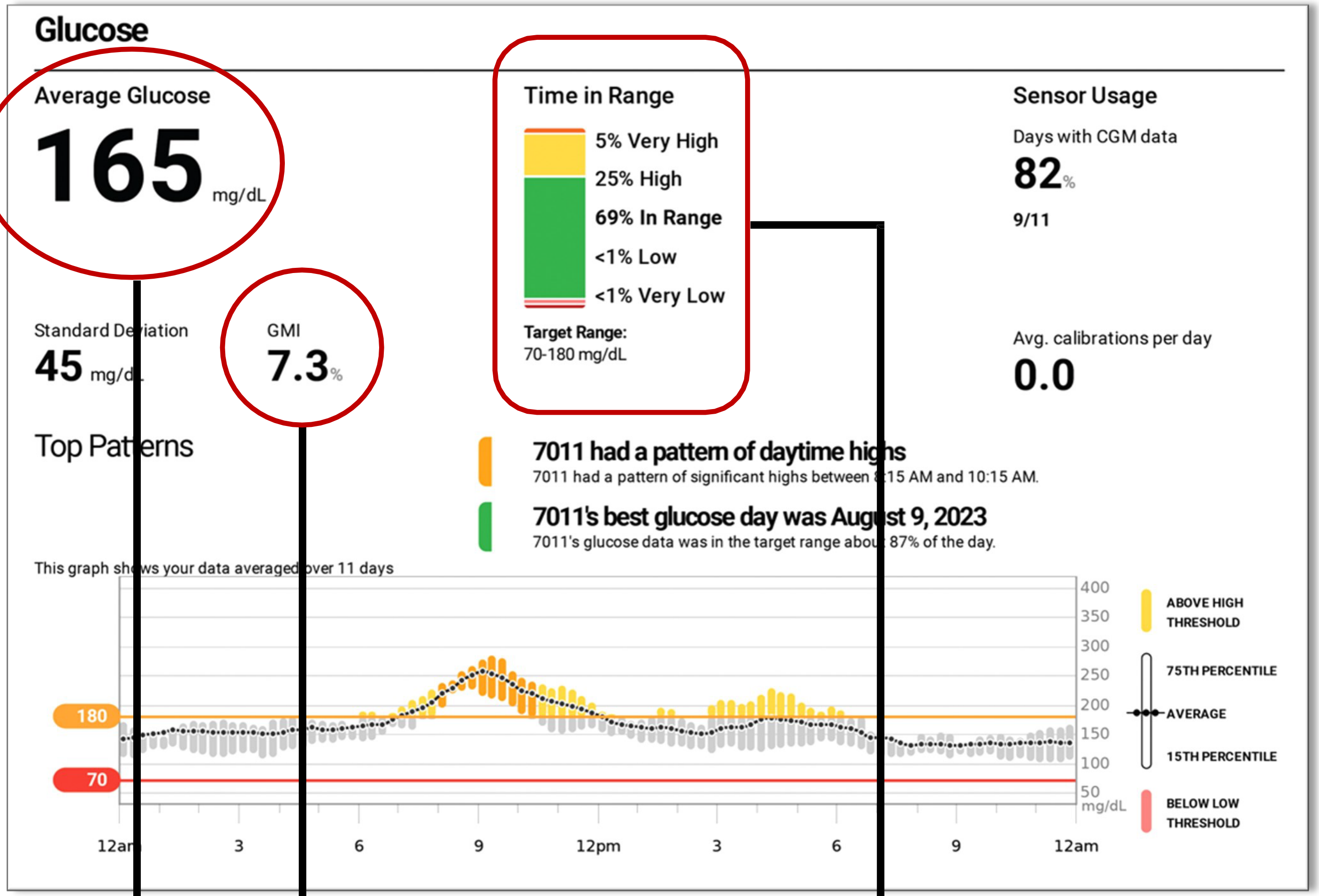

Average Glucose mg/dL	GMI* (estimated A1C) %
100	5.7
150	6.9
200	8.1
250	9.3
300	10.5

The "Time in Range" graph shows the percent of time your blood glucose levels were "low" or "very low" (less than or equal to 70 mg/dL). If you had Low or Very Low blood glucose greater than 4% of the time, please see a heath care provider as soon as possible.

For values in the RED range, please see a health care provider as soon as possible!

**Glucose Management Indicator (GMI)*
You may find your GMI value on page 3 of your CGM Report

medRxiv preprint doi: https://doi.org/10.64898/2026.03.30.26349552; this version posted April 4, 2026. The copyright holder for this preprint (which was not certified by peer review) is the author/funder, who has granted medRxiv a license to display the preprint in perpetuity.
It is made available under a CC-BY 4.0 International license .

APPENDIX E: Retinal Imaging Checklist

Participant ID: ☐ ☐ ☐ ☐ Visit: 1 VA: OD____________ OS____________

AI-READI: Imaging

Assessment Date: ___ ___/ ___ ___/ ___ ___ ___ ___ (mm dd yyyy) **Examiner Initials:**___________ **YOB:**___________

OD		OS	
start with OptoMed			
1) *OptoMed*-Disc centered-CFP	Y / N	2) *OptoMed*-Disc centered-CFP	Y / N
3) *OptoMed*-Mac centered-CFP	Y / N	4) *OptoMed*-Mac centered-CFP	Y / N
put OptoMed camera back on charger and move to Eidon **DILATE the PARTICIPANT in BOTH EYES** ***the required pupillary dilation for imaging on the *Eidon* is 3.0mm***			
5) *Eidon*-UWF Central-IR	Y / N	7) *Eidon*-UWF Central-IR	Y / N
6) *Eidon*-UWF Central-FAF	Y / N	8) *Eidon*-UWF Central-FAF	Y / N
switch to SmartMosaic mode			
9) *Eidon*-UWF Central-CFP	Y / N	13) *Eidon*-UWF Central-CFP	Y / N
10) *Eidon*-UWF Nasal-CFP	Y / N	14) *Eidon*-UWF Nasal-CFP	Y / N
11) *Eidon*-UWF Temporal-CFP	Y / N	15) *Eidon*-UWF temporal-CFP	Y / N
12) *Eidon*-Create Mosaic Image-CFP	Y / N	16) *Eidon*- Create Mosaic Image-CFP	Y / N
move to Spectralis			
17) *Spec*-ONH-RC-HR-OCT	Y / N	18) *Spec*-ONH-RC-HR-OCT	Y / N
19) *Spec*-PPole Mac-HR-61 lines-OCT	Y / N	20) *Spec*-PPole Mac-HR-61 lines-OCT	Y / N
21) *Spec*-Mac-20x20-HS-512 lines-OCTA	Y / N	22) *Spec*-Mac-20x20-HS-512 lines-OCTA	Y / N
move to Cirrus			
23) *Cirrus*-Mac Cube-512x128-OCT	Y / N	24) *Cirrus*-Mac Cube-512x128-OCT	Y / N
25) *Cirrus*-Disc Cube-200x200-OCT	Y / N	26) *Cirrus*-Disc Cube-200x200-OCT	Y / N
27) *Cirrus*-Macula 6x6-OCTA	Y / N	28) *Cirrus*-Macula 6x6-OCTA	Y / N
29) *Cirrus*-Disc 6x6-OCTA	Y / N	30) *Cirrus*-Disc 6x6-OCTA	Y / N
move to Maestro-2			
31) *M2*-3D Wide(H) 12x9-512x128-OCT	Y / N	32) *M2*-3D Wide(H) 12x9-512x128-OCT	Y / N
33) *M2*-3D Macula 6x6-512x128-OCT	Y / N	34) *M2*-3D Macula 6x6-512x128-OCT	Y / N
35) *M2*-Mac 6x6-360x360-(rep3)-OCTA	Y / N	36) *M2*-Mac 6x6-360x360-(rep3)-OCTA	Y / N
move to Triton			
37) *Triton*-3D(H)+Rad 12x9-512x256-OCT	Y / N	38) *Triton*-3D(H)+Rad 12x9-512x256-OCT	Y / N
39) *Triton*-Macula 6x6-320x320-OCTA	Y / N	40) *Triton*-Macula 6x6-320x320-OCTA	Y / N
41) *Triton*-Macula 12x12-512x512-OCTA	Y / N	42) *Triton*-Macula 12x12-512x512-OCTA	Y / N
move to FLIO			
43) *FLIO*-Mac-HS	Y / N	44) *FLIO*-Mac-HS	Y / N

Comments:

____

____

____

medRxiv preprint doi: https://doi.org/10.64898/2026.03.30.26349552; this version posted April 4, 2026. The copyright holder for this preprint (which was not certified by peer review) is the author/funder, who has granted medRxiv a license to display the preprint in perpetuity. It is made available under a CC-BY 4.0 International license .

Your AI-READI Test Results

Thank you for participating in the AI-READI study.

As part of the study, we performed tests on your blood and urine. These tests help our assessment of your health status.

Below, we provide the result for some of these tests. In some cases, these tests may return a value outside of the test's established normal range. When this occurs, it does not mean that the participant is unhealthy or has an issue affecting their health, such as an illness. Instead, it simply means that the participant should share these test results with their healthcare provider.

If you have one or more test results that are outside of their normal range, please share them with your healthcare provider. Upon review, they will tell you whether more attention is required, such as repeating one or more of these tests.

Lab Test Overview

To assist in the interpretation of your test results, please review this visual explanation.

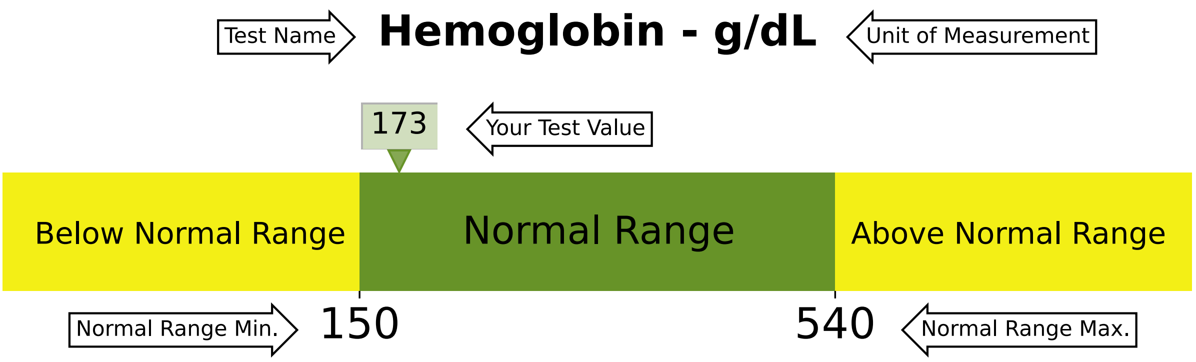

Explanation of Lab Tests

Glucose: This test checks how much sugar is in your blood. If it's too high, it might mean your body isn't using sugar properly, which could be due to diabetes. It could also be high if you have eaten recently.

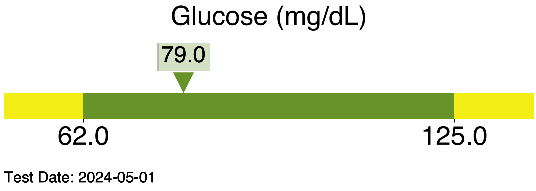

HbA1c: This test shows your average blood sugar levels over the past 2 to 3 months. It can be used to diagnose diabetes. If you have been diagnosed with diabetes, it can also help indicate whether your diabetes is under control.

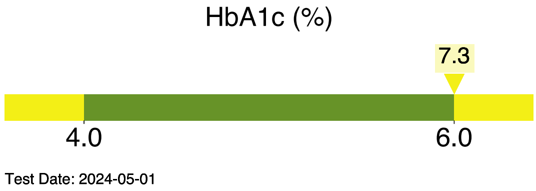

medRxiv preprint doi: https://doi.org/10.64898/2026.03.30.26349552; this version posted April 4, 2026. The copyright holder for this preprint (which was not certified by peer review) is the author/funder, who has granted medRxiv a license to display the preprint in perpetuity.
It is made available under a CC-BY 4.0 International license .

C-Peptide: This test measures how much insulin your body is making. Insulin helps control blood sugar.

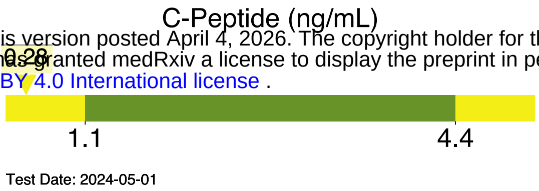

Insulin: This test checks how much insulin is in your blood. In type 2 diabetes, you might have too much insulin at first but less later on. Your insulin could also be high if you take insulin shots.

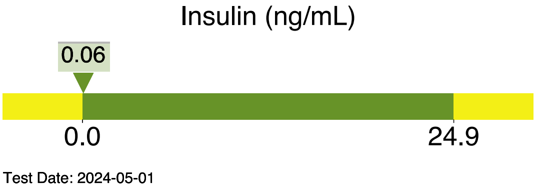

Total Cholesterol, Triglycerides, HDL Cholesterol, LDL Cholesterol: These tests look at different kinds of fats in your blood. If you have diabetes, you need to keep track of these fats to lower your risk of heart problems. The most important one is LDL-cholesterol.

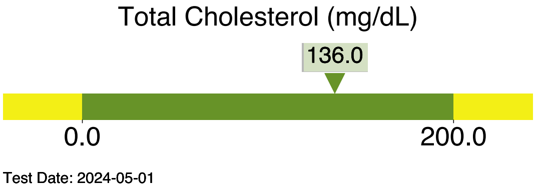

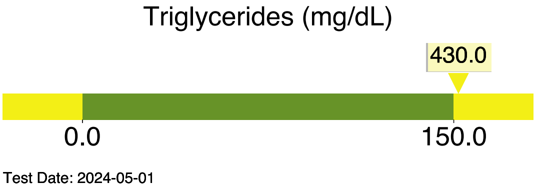

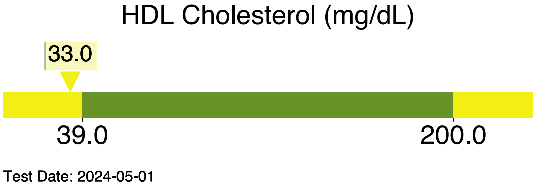

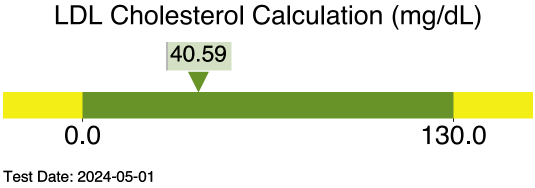

CRP-HS: This test checks for inflammation, which can be linked to diabetes and heart problems. High levels mean more inflammation.

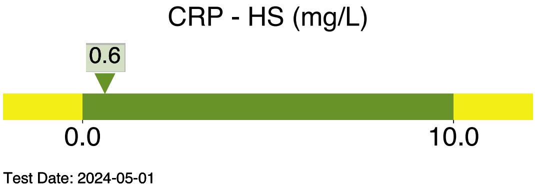

BUN, Creatinine: These tests look at how well your kidneys are working. Diabetes can sometimes hurt your kidneys, so these tests help to keep track.

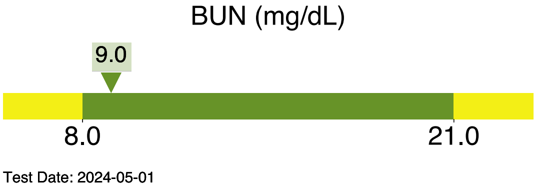

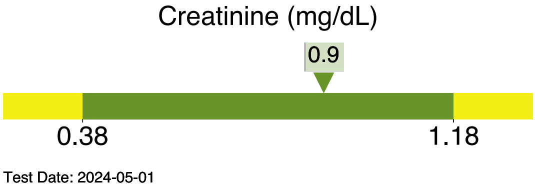

Sodium, Potassium, Chloride, Carbon Dioxide (Total CO2): These tests measure important salts and gasses in your blood. They help check if everything is balanced, which is important for managing diabetes.

medRxiv preprint doi: https://doi.org/10.64898/2026.03.30.26349552; this version posted April 4, 2026. The copyright holder for this preprint (which was not certified by peer review) is the author/funder, who has granted medRxiv a license to display the preprint in perpetuity.
It is made available under a CC-BY 4.0 International license .

Sodium (mEq/L)

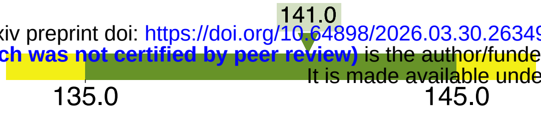

Test Date: 2024-05-01

Potassium (mEq/L)

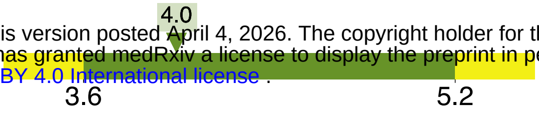

Test Date: 2024-05-01

Chloride (mEq/L)

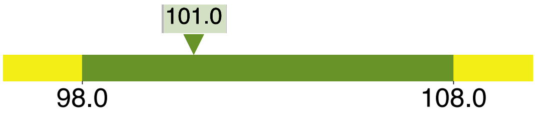

Test Date: 2024-05-01

Carbon Dioxide, Total (mEq/L)

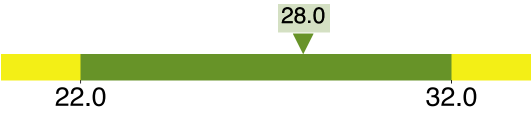

Test Date: 2024-05-01

Calcium, Total Protein, Albumin: These tests give information about your overall health and can show if diabetes is affecting other parts of your body.

Calcium (mg/dL)

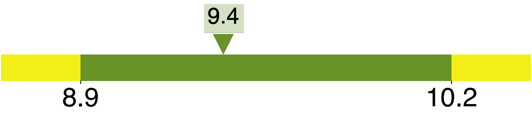

Test Date: 2024-05-01

Protein, Total (g/dL)

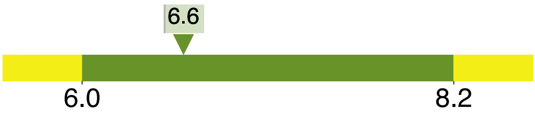

Test Date: 2024-05-01

Albumin (g/dL)

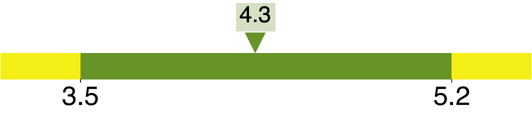

Test Date: 2024-05-01

Urine Albumin/Creatinine Ratio (uACR): This test checks your urine to see if your kidneys are being affected by diabetes. It can help catch kidney problems early. Note that menstrual periods, infection, and exercise can lead to falsely high urine albumin levels, which can affect its intepretation.

Urine Albumin/Creatinine Ratio (mg/g)

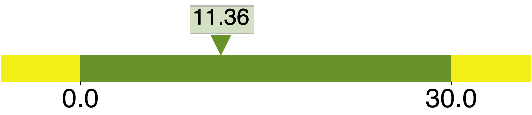

Test Date: 2024-05-01

Bilirubin, Alkaline Phosphatase, AST, ALT: These tests check how well your liver is working. Diabetes and some diabetes medicines can affect the liver.

Bilirubin, Total (mg/dL)

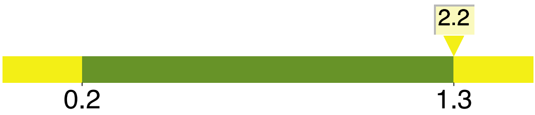

Test Date: 2024-05-01

Alkaline Phosphatase (IU/L)

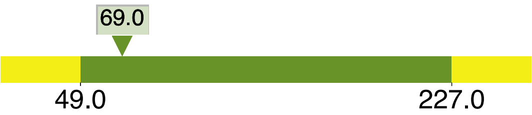

Test Date: 2024-05-01

AST (IU/L)

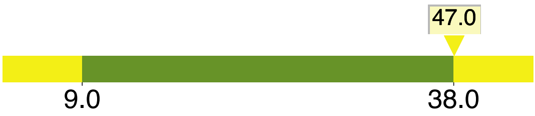

Test Date: 2024-05-01

ALT (IU/L)

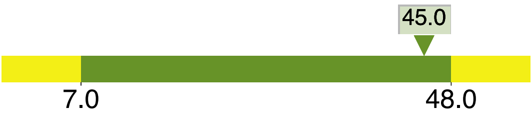

Test Date: 2024-05-01

N-terminal pro-B-type natriuretic peptide, Troponin-T: These tests check if your heart might be damaged, which is important because people with diabetes have a higher risk of heart problems.

medRxiv preprint doi: https://doi.org/10.64898/2026.03.30.26349552; this version posted April 4, 2026. The copyright holder for this preprint (which was not certified by peer review) is the author/funder, who has granted medRxiv a license to display the preprint in perpetuity.
It is made available under a CC-BY 4.0 International license .

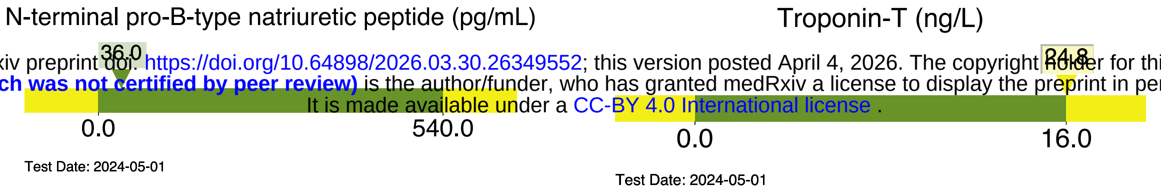

Complete Blood Count (CBC): This test looks at different parts of your blood. It helps doctors see if you are healthy or if something might be wrong. Here are some things the CBC test checks:

- **Red Blood Cells (RBCs):** These cells carry oxygen from your lungs to the rest of your body. If you have too few, you might feel tired because your body isn't getting enough oxygen.
- **White Blood Cells (WBCs):** These cells help fight off germs and keep you from getting sick. If you have too many or too few, it could mean your body is fighting an infection, or there might be another problem.
- **Platelets:** These tiny cells help your blood to clot. When you get a cut, platelets help stop the bleeding. If you don't have enough platelets, you might bleed more than usual.
- **Hemoglobin:** This is a part of red blood cells that carries oxygen. Low levels can make you feel very tired and weak.
- **Hematocrit:** This measures how much space red blood cells take up in your blood. It helps doctors understand if you have enough red blood cells

Additional parts of this test that your doctor can explain if needed are: **MCV (Mean Corpuscular Volume)**, **MCH and MCHC (Mean Corpuscular Hemoglobin and Concentration)** and **RDW (Red Cell Distribution Width)**. These tests help determine different types of low blood counts.

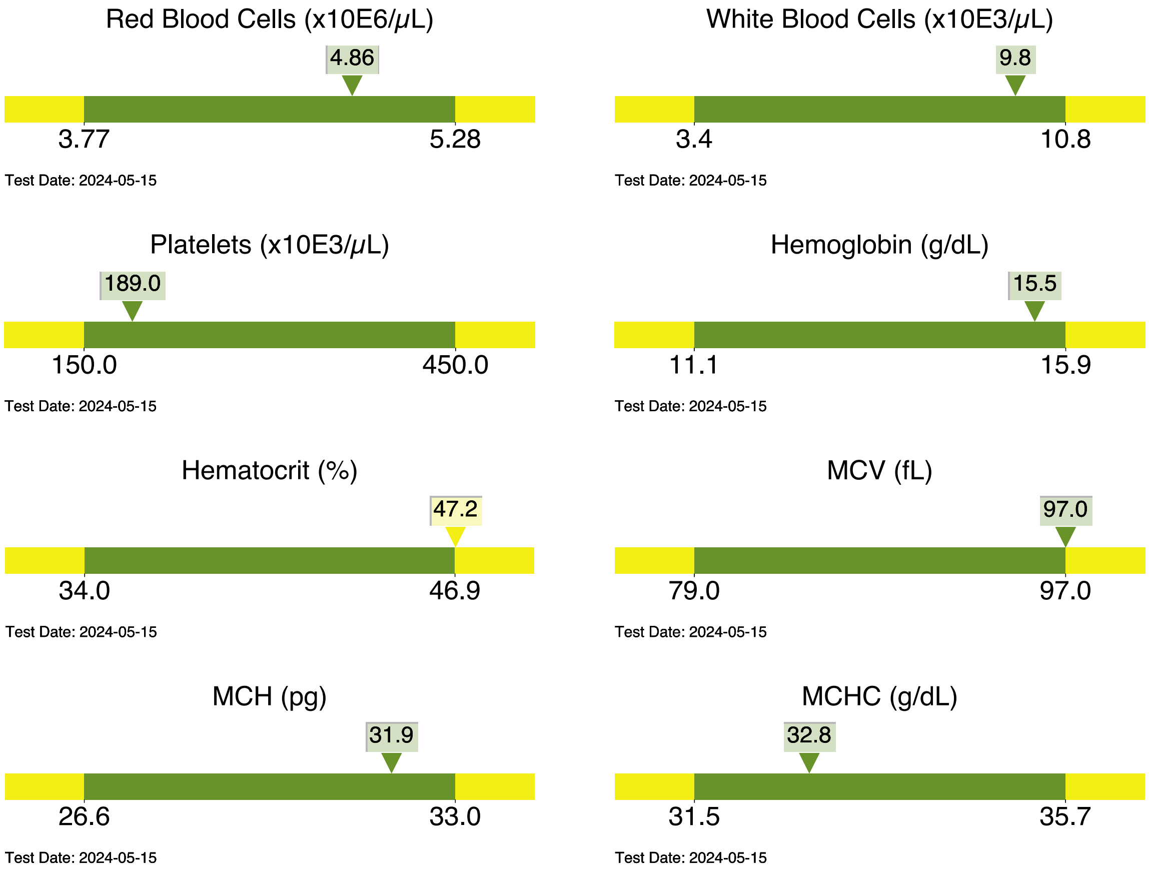

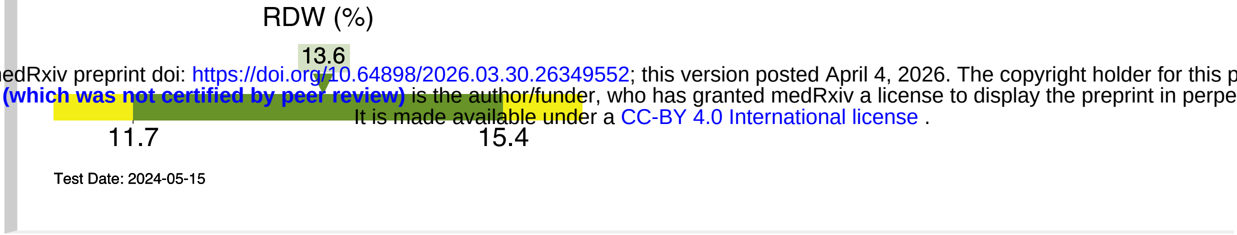

medRxiv preprint doi: https://doi.org/10.64898/2026.03.30.26349552; this version posted April 4, 2026. The copyright holder for this preprint (which was not certified by peer review) is the author/funder, who has granted medRxiv a license to display the preprint in perpetuity.
It is made available under a CC-BY 4.0 International license .

medRxiv preprint doi: https://doi.org/10.64898/2026.03.30.26349552; this version posted April 4, 2026. The copyright holder for this preprint (which was not certified by peer review) is the author/funder, who has granted medRxiv a license to display the preprint in perpetuity.
It is made available under a CC-BY 4.0 International license .

Exit Survey

Screening ID 35539-V3

How clear were the website instructions provided at the beginning of the study?

- Very clear
- Clear
- Neutral
- Somewhat clear
- Very unclear

When reviewing multiple pages of study materials online, did you experience any difficulty navigating the documents?

- Always
- Often
- Sometimes
- Rarely
- Never

Were the tasks and activities in the study easy to understand (completing the survey, wearing the devices, mailing devices back)?

- Very easy to understand
- Easy to understand
- Neutral
- Difficult to understand
- Very difficult to understand

How did you feel about the time involved during the data collection for this study?

- Very satisfied
- Satisfied
- Neutral
- Dissatisfied
- Very dissatisfied

How would you rate the overall communication with the study team?

- Very good
- Good
- Neutral
- Poor
- Very poor

How would you rate your overall experience with the in-person data collection visit?

- Very good
- Good
- Neutral

medRxiv preprint doi: https://doi.org/10.64898/2026.03.30.26349552; this version posted April 4, 2026. The copyright holder for this preprint (which was not certified by peer review) is the author/funder, who has granted medRxiv a license to display the preprint in perpetuity.
It is made available under a CC-BY 4.0 International license .

- ◯ Somewhat poor
- ◯ Very poor

How would you rate your overall experience with the at-home data collection component of the study?

- ◯ Very good
- ◯ Good
- ◯ Neutral
- ◯ Somewhat poor
- ◯ Very poor

After completing the study, how did your feelings about your participation compare to your initial expectations?

- ◯ Very similar, met expectations
- ◯ Somewhat similar, somewhat met expectations
- ◯ Neutral
- ◯ Somewhat different, somewhat met expectations
- ◯ Very different, did not meet expectations

Would you recommend participation in this study to others?

- ◯ Strongly agree
- ◯ Agree
- ◯ Neutral
- ◯ Somewhat disagree
- ◯ Strongly disagree

Please provide any additional comments or feedback you may have related to the questions in this survey in the textbox below. Write the number of the question next to your comments. Please note there is enough space so you can address several questions if needed.

Form Status

Complete? Incomplete

-- Cancel --
